# Medium Chain Triglycerides enhances exercise endurance through the increased mitochondrial biogenesis and metabolism

**DOI:** 10.1371/journal.pone.0191182

**Published:** 2018-02-08

**Authors:** Ying Wang, Zhenzhen Liu, Yi Han, Jiping Xu, Wen Huang, Zhaoshen Li

**Affiliations:** Changhai Hospital,Second Military Medical University, Yangpu District, Shanghai, China; State University of New York, UNITED STATES

## Abstract

Medium Chain Triglycerides (MCT) is a dietary supplement and usually used along with medications for treating food absorption disorders including diarrhea, steatorrhea and liver disease. It has been shown that MCT plays a role in lowering weight, and decreasing metabolic syndrome, abdominal obesity and inflammation. However, it is still unknown whether MCT enhances exercise endurance. Here, we demonstrated that MCT containing diet improves high temperature induced exercise performance impairment. We found that MCT up-regulates the expression and protein levels of genes involved in mitochondrial biogenesis and metabolism. Further investigation demonstrated that the increased mitochondrial biogenesis and metabolism is mediated through the activation of Akt and AMPK signaling pathways and inhibition of TGF-β signaling pathway. Collectively, our findings indicate a beneficial effect of dietary MCT in exercise performance through the increase of mitochondrial biogenesis and metabolism.

## Introduction

Medium-chain triglycerides (MCT) is triglycerides composed of a glycerol backbone and three fatty acids that have an aliphatic tail of 6–12 carbon atoms. MCT usually has 2 or 3 of the fatty acid chains belong to medium-chain fatty acids (MCFA). MCT exists in natural foods with high percentage found in coconut oil, palm kernel oil, desiccated coconut, and raw coconut meat according to the US Department of Agriculture National Nutrient Database. Unlike long chain triglycerides (LCT), MCT will be broken down into glycerol and MCFAs, which will be directly absorbed into the blood stream and thereby transported to the target organs [1].

The effects of MCT in energy expenditure, food consumption, and fat deposition have been well investigated [2]. Though inconsistence exists, a number of studies support the use of MCT as a supplement for weight loss [3]. Studies in both human and rodents have shown that oxidation of MCFA that hydrolyzed from MCT induces weight loss through increasing energy expenditure and fat oxidation and helping in the process of excess calorie burning [2,4,5]. Baba et al showed that MCT fed rats have less body weight, lower fat accumulation, smaller size of adipocytes and higher basal and stimulated metabolic rates [6]. Rothwell and colleagues demonstrated that except for the elevated rates of energy expenditure, the reduction in weight gains of animals fed with MCT were possibly due to sympathetic activation of brown fat thermogenesis [7]. Furthermore, addition of the combination of chilli and MCT to meals increased diet-induced thermogenesis by over 50% in heathy normal-weight humans [8].

Human and rodent studies have shown that MCT also play a role in food intake and satiety [9]. A human study conducted in overweight men demonstrated that MCT consumption reduced food intake acutely in a glucagon-like peptide (GLP)-1, peptide YY (PYY) and insulin independent pathway [10]. Energy intake in normal-weight men at lunch was significantly lower after the MCT-containing breakfast than after all other breakfasts in a clinical trial of establishing the influence of chain length and satiety [11]. Bray et al. observed that rats fed with diets containing MCT consume less food compared with those fed with long chain triglycerides (LCT) containing food [12].

While health benefits from MCT seem to occur, the role of MCT in muscle function and exercise performance has not been established yet [9]. Here, we provide the first hand evidence that MCT improves heat induced deficiency in exercise performance in mice through the activation of glucose and lipid metabolism pathways and mitochondrial biogenesis in the skeletal muscles.

## Materials and methods

### Animals

Male ICR mice (12-week-old) were purchased from Center of experimental animals, Second Military Medical University (Shanghai, China). Animals were housed at ambient temperature (23–25°C) under a standard 12-h light-dark cycle (lights on at 7 AM) with access to food and water ad libitum. Mice were randomly divided into 3 groups (n = 10) and fed with regular chow (containing 20% protein, 70% carbohydrate and 10% of fat) or MCT diet (with 11.6g/kg of MCT added to the regular chow) for 7 days before training started.

### Exercise studies

For normal temperature exercise, mice were trained on the treadmill at a speed of 14 m/min daily for a period of 2 weeks. Mice were also trained on rotating rod (rotarod) for 15 min (speed 16-20r/min at beginning and increased to 32r/min thereafter) at day 1 and 8. For the high temperature exercise study, mice were accommodated at high temperature chamber (32°C) for 1 hour followed by 1 hour of exercise in the chamber at day 1–3. The temperature was increased to 34°C in day 4–6 and further increased 36°C in day 7–14. The exercise was achieved at a speed of 14 m/min on the treadmill. At day 1 and 8, mice were subjected to a rotarod training for 15 min using the same protocol as in normal temperature groups. On day 15, all mice were run on the treadmill for 1 hour followed by rotarod until exhaustion. The running time for each mouse on rotarod was recorded. The study protocol was reviewed and approved by the Institutional Animal Care and Use Committees of the Second Military Medical University.

### RNA extraction and real-time PCR

Skeletal muscle total RNA was extracted using the Trizol (Invitrogen). First-strand cDNA was synthesized from 1 μg of RNA (M-MLV First Strand cDNA Synthesis Kit; Tiangen Biotech, Beijing, China) according to the manufacturer's instructions. Real-time PCR was carried out in a 10-μl reaction mixture (SYBR green master Mix; Life Tech) containing 1 μl of the diluted (1:10) first-strand cDNA, and the results were normalized to *actin* using ΔΔCT. The sequences of the primers used for amplification of mouse tfam (mitochondrial transcription factor A), uqcrc (Ubiquinol Cytochrome c Reductase), cox5b (Cytochrome c oxidase 5B) *pgc-1a* (*peroxisome proliferative activated receptor gamma coactivator 1 alpha)*, *ATP5A (mitochondrial membrane ATP synthase)*, tgf-*β (transforming growth factor beta)*, *smad3* and *actin* are listed in S1 Table.

### Immunoblotting

Skeletal muscles were homogenized in RIPA buffer (50 mM Tris, pH 7.4, 150 mM NaCl, 1% Triton X-100, and 0.1% SDS) containing proteinase inhibitors (Roche). Twenty micrograms of total protein was resolved on 10% SDS-PAGE and then transferred to polyvinylidene fluoride membranes. The membranes were blocked with 5% non-fat dry milk in Tris-buffered saline with Tween 20 (TBS-T) for 1 h at room temperature. Antibodies (protein kinase B/Akt, p-Akt Ser473, AMP-activated protein kinase (AMPK), pAMPK, Smad3 and β-Actin were from Cell Signaling Technologies; mTOR, p-mTOR, TGF-β and ATP-5a were purchased from Abcam; and PGC-1α was obtained from Santa Cruz) diluted in TBS-T containing 5% of BSA were added to the membranes and incubated overnight at 4°C. The membranes were washed three times with TBS-T and incubated with horseradish peroxidase (HRP)-conjugated secondary antibodies for 1 h at room temperature. Signals detected using the SuperSignal West Dura extended the duration substrate (Thermo Scientific, Rockford, IL).

### Statistical analysis

All reported data are presented as means ± standard error of the mean (SEM). When comparing two groups between control and MCT, independent two-tailed t-test was used. Dynamic comparison for body weight, GraphPad 5.0 two-way ANOVA (group × time) was used. For each statistically significant F value observed for the main effect or interaction, a two-tailed post hoc test (Tukey's) was applied to determine individual differences between means. Differences were considered to be statistically significant at P ≤ 0.05 for primary outcomes and P ≤ 0.01 for secondary outcomes.

## Results

### MCT does not affect body weight and food intake

**Fig 1:**
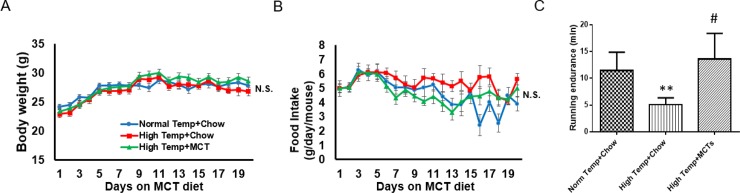
**Body weight (A), food intake (B) and running endurance (C) of mice** fed with chow diet or MCT containing diet under normal and high temperature conditions. N.S. no significant difference, # p<0.05, ** p<0.01.

Twelve-week-old ICR mice were fed with regular chow with or without the supplemental of MCT for 21 days. The mice were also trained for exercise under room temperature or high temperature in the last 14 days. Our data demonstrated that MCT feeding does not affect body weight (Fig 1A) and food intake (Fig 1B) under high temperature condition. We also found that the levels blood glucose and serum triglycerides (TG) are not changed by MCT (data not shown).

### MCT enhances exercise performance under high temperature condition

To assess the effect of MCT on exercise performance, we trained the mice for exercise using treadmill for 14 days under normal or high temperature. We then evaluated the running endurance of the mice using rotarod performance test. We showed that the exercise performance is significantly decreased in high temperature environment, while mice fed with MCT containing diet have much better performance compared to the regular chow diet fed mice (Fig 1C). We observed a more than 2-fold increase in running time that under high temperature. This demonstrated a rescue of heat induced deficiency in exercise and possibly skeletal muscle function by MCT.

### MCT increases mitochondrial biogenesis and metabolism

**Fig 2:**
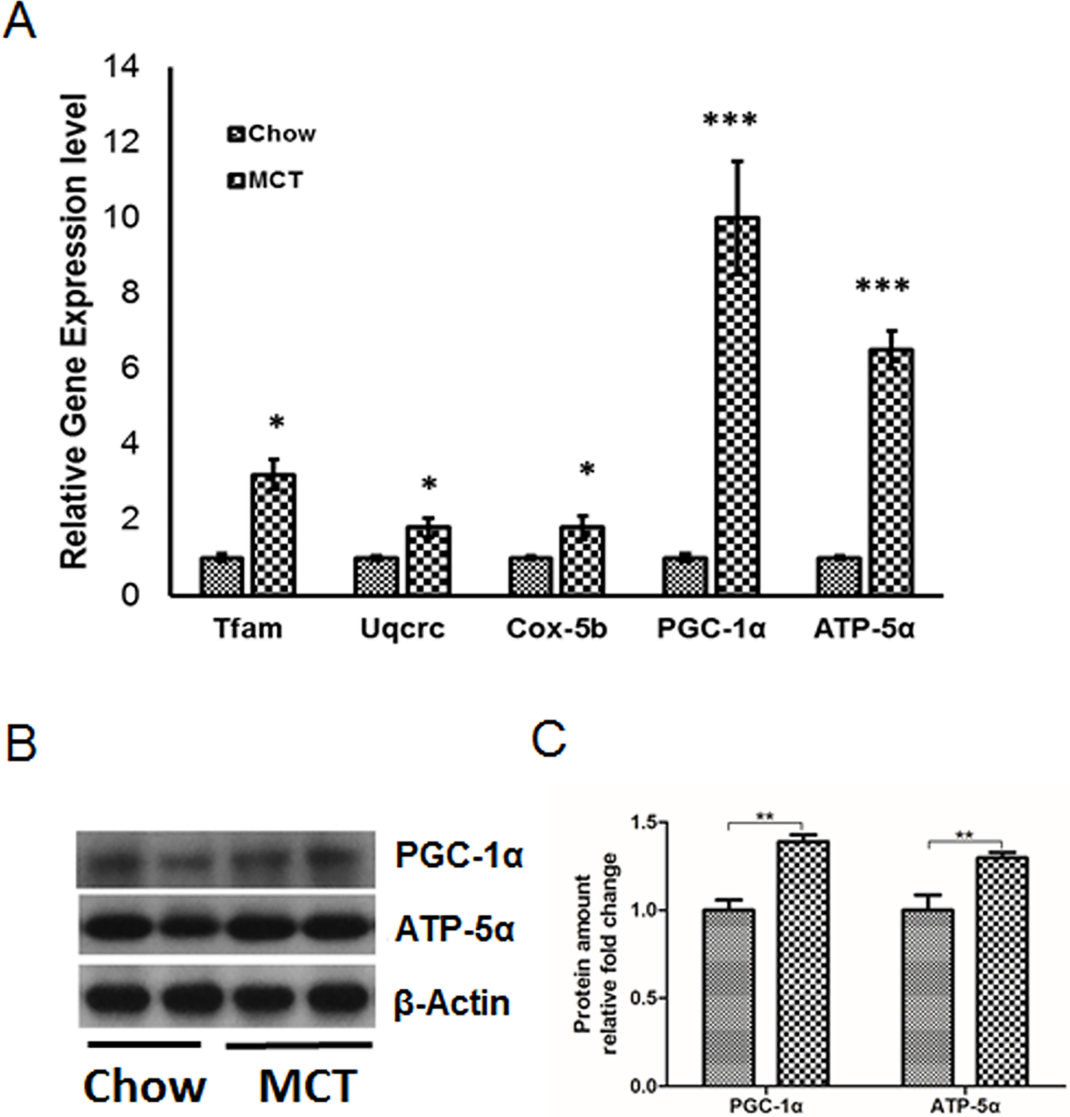
MCT increases mitochondrial biogenesis and metabolism under high temperature. Gene expression (A) and protein (B) levels for mitochondrial biogenesis and metabolism markers in the skeletal muscle. C: quantification of the immunoblot. ** p<0.01, *** P<0.001.

Mitochondria are the primary energy source for skeletal muscle contraction. To elucidate if the increased exercise performance by MCT in mice could be a result of increased mitochondrial mass and function, we evaluated the levels of mRNA and protein that involved in mitochondrial biogenesis and respiratory chain. As shown in Fig 2A, gene expression levels of *tfam* (mitochondrial transcription factor A), a marker of mitochondrial biogenesis, *uqcrc* (Ubiquinol Cytochrome c Reductase), a respiratory chain protein on complex III, *cox5b* (Cytochrome c oxidase 5B), the last enzyme in the mitochondrial electron transport chain in complex IV, peroxisome proliferative activated receptor gamma coactivator 1 alpha (PGC-1α), a key regulator of energy metabolism and mitochondrial membrane ATP synthase (ATP5α) were significantly upregulated by MCT. We also observed a significant increase in protein level of PGC-1α and ATP5α (Fig 2B and 2C). These indicate that MCT improves skeletal muscle function through the induction of mitochondrial biogenesis and metabolism under high temperature condition.

### MCT activates Akt and AMPK signaling pathways

**Fig 3:**
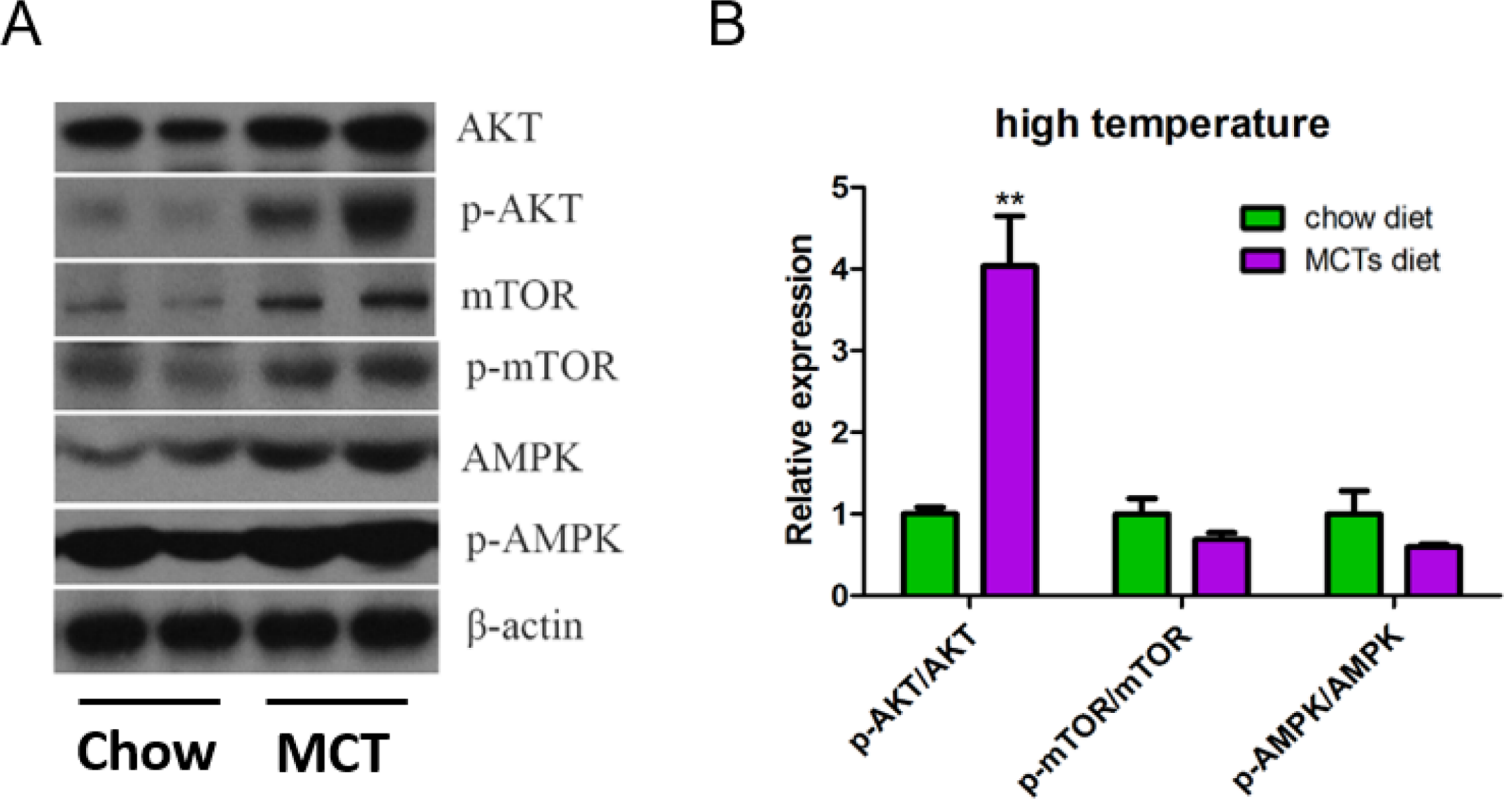
MCT activates Akt and AMPK signaling pathways under high temperature. Immunoblot (A) and quantification (B) in skeletal muscle. ** p<0.01.

Mitochondrial biogenesis and metabolism are regulated by many signaling pathways including Akt and AMPK signaling, the best-characterized protein kinases known to target PGC-1α. To elucidate whether the increased mitochondrial biogenesis and metabolism by which MCT are mediated through the activation AKt and AMPK pathways, we isolated the skeletal muscle (quadriceps) from the mice and analyzed the metabolic pathways. Indeed, we found that Akt signaling is activated by MCT (Fig 3A and 3B). This is demonstrated by increased levels in both total and phosphorylated Akt and mTOR (Fig 3A and 3B). The results also showed that MCT increases total and phosphorylated AMPK levels in the skeletal muscle. These demonstrate that the increased mitochondrial biogenesis and metabolism is mediated through the activation of Akt and AMPK signaling pathways.

### MCT downregulates TGF-β signaling

**Fig 4:**
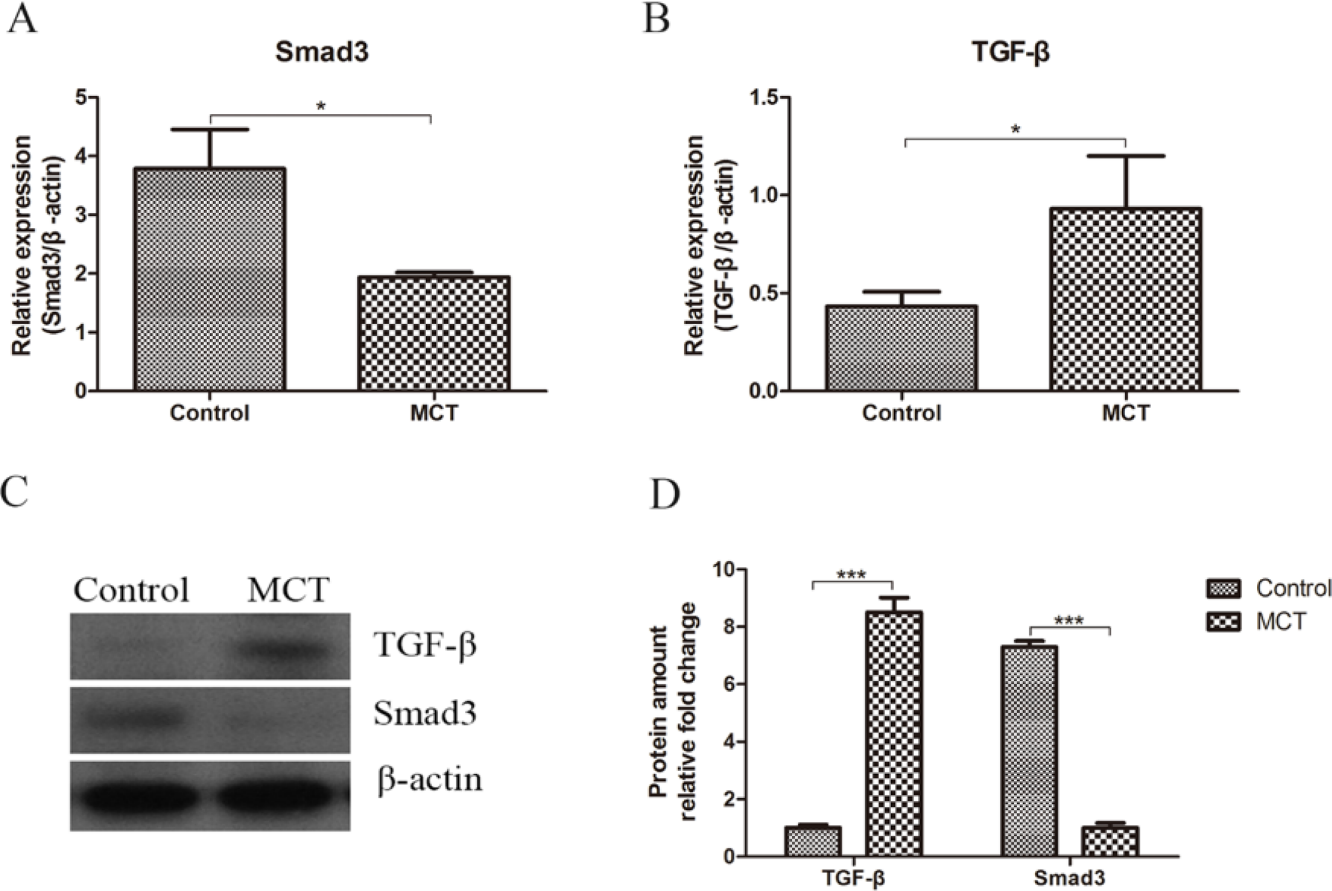
MCT inhibits TGF-β signaling pathway under high temperature. Gene expression levels of Smad3 (A) and TGF-β (B); C and D: Immunoblot and quantification for Smad3 and TGF-β. * p<0.05, *** p<0.001.

The mitochondrial biogenesis and metabolism are also negatively controlled by TGF-β signaling. To elucidate whether MCT also controls TGF-β signaling, we performed real time PCR and immunoblot studies using the skeletal muscle tissue, and we found that both gene expression (Fig 4A) and protein (Fig 4B and 4D) Smad3, a key mediator of TGF-β signaling are inhibited by MCT. We also observed an upregulation of TGF-β in both mRNA (Fig 4C) and protein (Fig 4B and 4D) levels. We think that this is a compensatory response of the huge reduction in Smad3.

## Discussion

**Fig 5:**
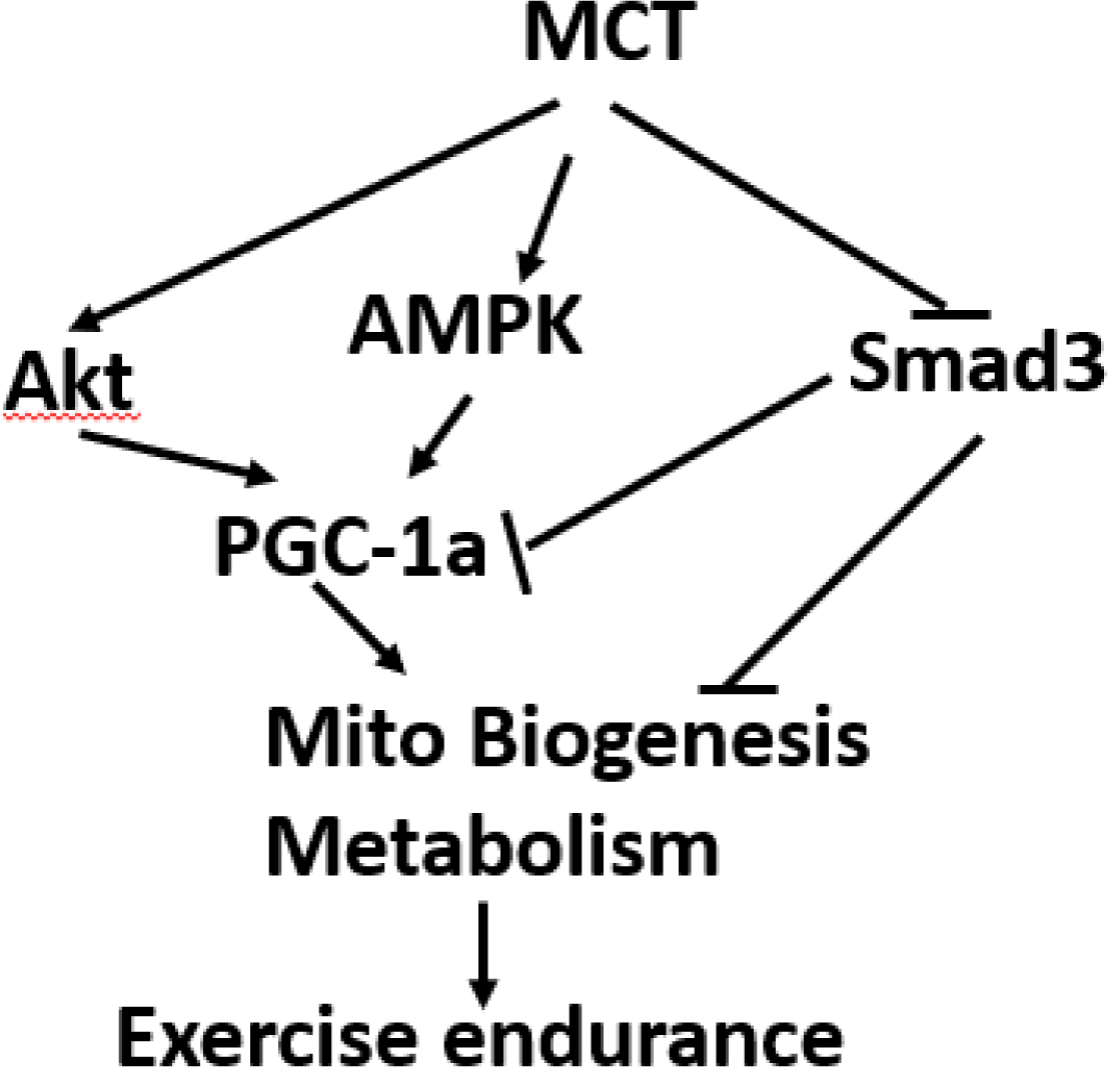
Schematic illustration of the effects of MCT in exercise.

In studies presented here, we demonstrated that MCT improves high temperature induced defect in exercise performance and skeletal muscle function in mice. This was also coupled with the activation of Akt and AMPK signaling pathways, inhibition of TGF-β signaling and subsequent increase in mitochondrial biogenesis and metabolism in the skeletal muscle (Fig 5).

Our results are consistent with recent findings on MCT, in combination with leucine and vitamin D, increase muscle strength and function in human [13]. The authors reported that the combination of MCT, but not LCT, leucine and vitamin D, increases body weight, right-hand grip strength, walking speed, leg open-and-close test performance and peak compare to the control group in a 3-month trial in frail elderly individuals [13]. However, because of the missing of MCT only group, the exact role of MCT in muscle strength and function could not be concluded [13]. It is reported that MCT does not provide beneficial effect to exercise performance under normal condition in humans [9,14,15], but it unclear if this is also true under stress condition, such as heat stress, when the exercise performance and capacity are impaired [16]. Thus, we investigated the effects of MCT on hot environments induced decrease in exercise capacity. Our study demonstrated that MCT preserves heat stress induced- exercise performance and capacity impairment in mice.

MCT has been reported to play a role in reducing body weight compared to LCT feeding group in both humans and rodents [17,18]. We showed that there is no significant differences between groups in body weight. This is consistent with the previous findings demonstrating that the significant body weight change only exists when comparing MCT feeding mice to LCT but not chow diet feeding mice [18]. We think that this may be due to 2 reasons: 1) a potential protective effect of MCT may exist to protect body against heat stress induced dehydration and weight loss; and 2) a potential increase in muscle mass. Further studies are needed to evaluate whether MCT supplement increases muscle mass especially under heat stress condition.

Another intriguing finding of the current investigation is the increased mitochondrial biogenesis and metabolism by MCT in vivo. Mitochondria are the key organelle for complete oxidation of glucose and fatty acid and ATP generation, the most critical energy source for muscle contraction. Thus, it is expected that the increased running endurance is coupled with the increased energy output from mitochondria, which could be due to the up-regulation of mitochondrial mass or enhanced mitochondrial metabolism. PGC-1α and Tfam are master regulators of mitochondrial biogenesis [19–21]. Increased levels of PGC-1α and Tfam in MCT treated mice suggests a positive role of MCT on mitochondrial biogenesis in skeletal muscle. On the other hand, exercise is also an independent regulator for mitochondrial biogenesis [22], thus, the increased mitochondrial biogenesis could also be a result of increased exercise performance by MCT. While enhancement in mitochondrial metabolism through upregulating of enzymes in different complexes in the mitochondria by MCT raises the question of an increased oxidative stress in the skeletal muscle, Montgomery and colleagues have demonstrated that MCT is associated with lower level of reactive oxygen species (ROS) in C2C12 muscle cells and mitochondria isolated from mouse muscle tissue [18]. They also observed less accumulation of oxidative damaged lipids and proteins in MCT treated group. In consistent with these reports, Saifudeen et al. also demonstrated that 4-month of treatment with 5% of MCT increases fatty acid oxidation, reduced hypertrophy and oxidative stress along with the maintenance of energy level in the heart in both 2- and 4-month-old rats [23]. These suggest that MCT promote mitochondrial metabolism without generating oxidative stress in the skeletal muscle.

Our mechanistic studies demonstrated that the increased mitochondrial biogenesis could be due to the activation of Akt and AMPK signaling pathways. These are also the major signaling pathways related to glucose and lipid metabolism in the skeletal muscles. Approximately 70–80% of glucose is taken up by skeletal muscle and is either stored as glycogen for future use or directly oxidized for energy [24]. The glucose uptake is regulated by insulin, insulin receptor and the downstream phosphorylation cascade. Akt is located at the heart of this cascade, and activation of Akt by phosphorylation leads to the translocation of glucose transporter 4 (GLUT4) to the plasma membrane to initiate glucose uptake. Phosphorylation of Akt is also an important indicator of insulin sensitivity of the skeletal muscle. We show in our study that MCT feeding significantly enhances phosphorylation level of Akt and its downstream molecule mTOR, suggesting that MCT increases insulin sensitivity and glucose utilization in the skeletal muscle, which is consistent with the studies performed in C2C12 cell line [18]. On the other hand, the total protein and phosphorylation form of AMPK, an enzyme that plays a role in cellular energy homeostasis are both increased in the skeletal muscle of MCT feeding mice. The activation of AMPK in skeletal muscle is coupled with the inhibition of lipogenesis, stimulation of fatty acid oxidation and muscle glucose uptake as well. Our data demonstrated that although the ratio of phosphorylation/total level of AMPK is not increased, both phosphorylated and total AMPK are elevated in the skeletal muscle. This indicates the increased phosphorylation comes from the increased total protein while its activation is not affected. Thus, we think that MCT affects neither the activity of upstream kinases of AMPK, such as the serine–threonine kinase liver kinase B1 (LKB1), nor the ratio of AMP to ATP in the skeletal muscle. Our findings on increased total AMPK and phosphorylation of AMPK in skeletal muscle by MCT implicate an increase in both lipid and glucose metabolism, but decrease in fatty acid synthesis. This also could be the molecular mechanism by which MCT increases energy expenditure and oxygen consumption reported by other groups *in vivo* [2,4] and *in vitro* [18]. Thus, it would be of great interest to study whether MCT regulates glucose and lipid metabolism through the activation of both Akt and AMPK signaling pathways in the skeletal muscle *in vivo*; and the underlying mechanisms by which MCT activates Akt and AMPK signaling pathways merit further investigations.

TGF-β signaling is required for normal tissue repair; however, excessive TGF-β signaling can lead to robust profibrotic gene expression in fibroblasts, resulting in tissue fibrosis [25]. It has been shown that TGF-β suppresses the expression of genes related to mitochondrial function including PGC-1α and NRF-2, ERR-α, and PPAR-γ [26]. TGF-β signaling modulates energy metabolism through controlling mitochondrial metabolism both directly and indirectly in different cell types [27]. TGF-β inhibits complex IV and mitochondrial respiration which in turn leads to increased ROS and decreased mitochondrial membrane potential associated with senescence in mink lung epithelial cells [28]. It is also reported that TGF-β negatively regulates UCP2, a mitochondrial uncoupling protein located in the inner mitochondrial membrane and facilitates energy dissipation as heat, therefore decreasing energy output from the mitochondria. [29]. Our data on inhibition of Smad3 in skeletal muscle by MCT feeding demonstrates the possibility of MCT increases ATP generation through the inhibition of TGF-β signaling and upregulation of PGC-1α gene expression. The increase in TGF-β gene expression and protein levels in MCT treated mice could be a feedback of the deficiency of its downstream signaling, which mimic the status of “TGF-β resistance”.

TGF-β signaling pathways also play essential roles in early embryonic development and in regulating tissue homeostasis [30]. Deregulated signaling has crucial roles in both the development of tumors and metastasis [31]. It is reported that TGF-β signaling pathways play dual roles in regulating tumor growth and metastasis. They suppress tumors and early carcinomas through the inhibition of cell cycle, induction of apoptosis and upregulation of immune response [32]. On the other hand, the suppressive effects of TGF-β on tumors may lost in many types of aggressive tumors, while tumor promoting and pro-invasive responses remain and prevail, leading to the development of distant metastases [32]. Furthermore, the expression TGFβ correlates with the stage of the tumor [33,34]; and blocking the TGFβ signaling pathway may provide unique therapeutic strategies for treating tumor metastasis [32]. Our data showed an increase in TGFβ level but decrease in Smad3 induced by MCT treatment, but it is still not clear if MCT plays a role in tumor through acting on TGFβ signaling pathway. Even though MCT has been used as a source ketogenic diet to facilitate cancer treatment by affecting tumor glucose metabolism and growth while maintaining the patient's nutritional status [35], its exact role in tumor suppression keeps largely unknow. Thus, more studies are need to elucidate the effects and underlying mechanisms of MCT in tumors.

In conclusion, our study provided the first evidence that MCT, as a food supplement, preserves high temperature induced impairment in exercise performance and muscle function. This could be mediated through the activation of muscle Akt and AMPK signaling pathways, inhibition of TGF-β signaling pathway and subsequent increase in mitochondrial biogenesis and metabolism in the skeletal muscle. Our study reveals a novel role of MCT in exercise, providing evidence for treating muscle dysfunction and exercise impairment in patients by taking MCT as food supplement.

## Supporting information

S1 Table Sequence of the primers. (DOCX)
