# Supplementary material for: Medium Chain Triglycerides enhances exercise endurance through the increased mitochondrial biogenesis and metabolism
Source: PLoS One. 2018 Feb 8;13(2):e0191182. doi: 10.1371/journal.pone.0191182 (PMC5805166; doi:10.1371/journal.pone.0191182)
Supplement: S1 Table — (DOCX) [file pone.0191182.s001.docx]

**Table 1: Sequence of the primers**

| Gene | Forward | Reverse |
| --- | --- | --- |
| tgf-β | GAAGGACCTGGGTTGGAAGT | AGGACCTTGCTGTACTGTGT |
| smad3 | GCTGAGTGCCTCAGTGACAGTG | GGTAGACAGCCTCAAAGCCCTG |
| pgc-1α | GTAAATCTGCGGGATGATGG | TCGTTCGACCTGCGTAAAG |
| atp5a1 | TGTGCGTCTGACCGAGTTGC | GATCTTGCTGGGCTCCAGTTTG |
| uqcrc2 | GCTGGAGAGGTTATCAATGCTGCC | TCAGGAAGCCCTCTGACGTCTCC |
| cox-5b | GGAGAGGGAGATCATGATAGCAGC | GTCCTCTTCACAGATGCAGCCC |
| tfam | TTCGGCTCAGGGAAAATTGAAGC | GTCTCCGGATCGTTTCACACTTC |
| β-actin | TGTCCACCTTCCAGCAGATGT | AGCTCAGTAACAGTCCGCCTAGA |
